# Patient Opinions about Virtual Consultations in Saudi Arabia: A Nationwide Cross-Sectional Study

**DOI:** 10.3390/healthcare12101001

**Published:** 2024-05-13

**Authors:** Saad Mohammed AlShareef, Abdullah Abdulaziz AlWabel

**Affiliations:** 1 Department of Medicine, College of Medicine, Imam Mohammad Ibn Saud Islamic University (IMSIU), P.O. Box 7544, Riyadh 13317, Saudi Arabia; 2 Seha Virtual Hospital, Ministry of Health, Riyadh 12382, Saudi Arabia; 3 King Saud University Medical City, King Saud University, Riyadh 12372, Saudi Arabia

**Keywords:** Kingdom of Saudi Arabia, satisfaction, telehealth, Telehealth Usability Questionnaire, virtual consultation

## Abstract

There have been no nationwide studies of patient opinions regarding telehealth in Saudi Arabia to identify the factors that might influence patients’ perceptions and satisfaction. This was a prospective cross-sectional study of adults in the general population who last engaged with a healthcare practitioner via a virtual appointment. The participants were recruited by convenience sampling across Saudi Arabia between November 2023 and January 2024, completing a questionnaire that gathered data on (i) basic demographic and virtual consultation information and (ii) telehealth service delivery and technology based on the Telehealth Usability Questionnaire. Of the 916 participants, 53.7% were female, with a mean age of 47.2 (14.1) years. Nearly half attended primary care appointments, with the remainder attending a range of hospital specialties. Over 90% preferred having a virtual appointment over an in-person visit. About half had telephone consultations, while about a third had video calls through hospital-provided platforms; >90% found virtual appointments useful and convenient, easy to use, effective, reliable, and produced a favorable clinical interaction; and 97.4% were satisfied with their remote consultation experience despite the technical interruptions. The individuals who were less happy with their virtual consultation were significantly younger, lived in urban areas, attended specialty clinics, were seen by a psychologist, preferred in-person appointments, and had consultations by telephone. These data provide momentum to continue with and expand telehealth, especially through video calls, supported by educational initiatives.

## 1. Introduction

There is a need for convenient, efficient, and accessible healthcare delivery to serve the needs of the growing population of the Kingdom of Saudi Arabia (KSA) (growth rate ~1.5%; population ~35 million people), three-quarters of whom are aged between 15 and 74 years [1,2]. About four-fifths of the population live in urban areas [2], and, like most advanced economies, >90% of people own smartphones and have internet access [3,4]. In the US and Europe, keeping pace with advances in technology, the effective implementation of telemedicine has enabled the broad adoption of the best healthcare practices [5]. While the adoption of telehealth across the Middle East has been slower than in some other high-income countries, hampered by cultural, financial, organizational, individual, technological, legal, and regulatory challenges [5], the KSA had begun to rapidly enhance the telemedicine service provision even before the COVID-19 pandemic. The KSA started to consider the potential of using telehealth as far back as 1990, and, in 2019, the KSA published new regulations on telemedicine that provided all clinical staff with a comprehensive framework on its use [6]. In the KSA, the Ministry of Health (MoH) delivers telemedicine through different platforms, such as outpatient telemedicine clinics (virtual clinics), 937 call centers, and the Sehhaty smartphone application supported by telemedicine training for healthcare professionals according to the global best practices [7]. These activities have been driven by robust evidence that telehealth delivers comparable quality and outcomes to traditional in-person visits [8] and have culminated in widespread telehealth use such that, in an eighteen-month period in 2021 and 2022, over a million virtual consultations were delivered to the population of the KSA [9].

However, it is not sufficient for telehealth to only compete favorably with in-person visits according to objective measures such as clinical outcomes [8] and value [10]. The successful implementation of telemedicine also relies on patient-related factors, such as the perceptions of and satisfaction with telehealth provision [11]. The drivers of the behavioral intention to use telehealth are complex and include several theoretical perspectives (e.g., the Technology Acceptance Model (TAM) [12], social capital theory [13], and social cognitive theory [14]). Indeed, integrating these three theoretical concepts into a cohesive framework showed that the social capital factors (social trust, institutional trust, and social participation) significantly positively affected the technological factors (perceived ease of use and usefulness), which influenced the telehealth use intention [15]. In practice, if the patients are not at least as satisfied with telehealth as they are with in-person visits, or if they have negative perceptions of the approach, they may refuse the option of telehealth, regardless of its benefits [11]. For instance, Woo et al. found that there was a high rate of telehealth refusal in heart failure patients at the point of referral, and, despite the patients feeling positive about the service, the reticence and refusal were driven by patient-related factors such as concerns over technology, ease-of-use, access to care, cost, and privacy [16]. In reality, systematic reviews of patient satisfaction with telemedicine have reported high levels of patient satisfaction with telemedicine [17,18,19,20] across the life spectrum, including in pediatric populations and their caregivers [21] and in older adults [22]. A very recent meta-analysis of over a hundred cross-sectional studies from around the world on patient satisfaction with telemedicine similarly reported very high patient satisfaction, with less than 3% reporting satisfaction levels below 75% [18]. The recent COVID-19 pandemic also prompted several studies on patient satisfaction with telemedicine in Saudi Arabia, reporting high satisfaction with telemedicine in both general [19,20,23,24,25] and specialist [26,27,28] practice.

Therefore, those patients not satisfied with telehealth may refuse to engage with it [11]. The patient satisfaction with telemedicine is high [17,18,19,20,29], including in specific cities or institutions in Saudi Arabia [19,20,23,24,25,26,27,28], but these studies may not be generalizable to the wider population. No study has comprehensively evaluated all the domains of usefulness, convenience, effectiveness, reliability, and satisfaction regarding virtual consultations, and there have been no nationwide studies regarding patients’ opinions about telehealth in Saudi Arabia to identify the factors that might influence patients’ perceptions and satisfaction. Identifying the factors that influence patients’ perceptions and satisfaction of telehealth is important to inform specific changes in service delivery or policy to improve the quality of the service provision, ensure equitable use, and prevent resource wastage.

Therefore, the purpose of this prospective, cross-sectional, longitudinal study was to address the problem of a lack of nationwide data on patient opinions about telehealth in the KSA and identify the demographic and service-related factors that might influence patients’ perceptions and satisfaction. To achieve this, we conducted a nationwide survey of those patients in the KSA who recently accessed health services via virtual consultations and, using an adapted version of the Telehealth Usability Questionnaire (TUQ) [30], assessed their opinions of the technology implementation and services across the six domains of telehealth usefulness and convenience, ease of use, effectiveness, reliability, quality of the interaction, and satisfaction. This research is important for practitioners and policymakers to support their continued use and development of telehealth supported by initiatives that target specific individual, cultural, and technological barriers.

## 2. Materials and Methods

This study is reported according to the STROBE statement for cross-sectional studies [31]. This was a prospective, cross-sectional, longitudinal study of adults in the general population (aged > 18 years) who last engaged with a healthcare practitioner via a virtual appointment, agreed to participate, and were competent to complete a questionnaire. The Institutional Review Board of Imam Mohammad Ibn Saud Islamic University approved the study protocol (reference number 588/2023). All participants provided written signed statements of informed consent.

Recruitment was carried out between November 2023 and January 2024 across KSA (central, western, eastern, northern, and southern regions). Participants were convenience-sampled in shopping malls, public gardens, university campuses, and in the streets by sixteen medical students, who were trained in the study objectives and the questionnaire. Questionnaires were completed by these medical students, who collected answers from participants after reading the questions to them. Recognizing the inherent bias in convenience sampling and the applicability of the results only to the population studied, we did not formally calculate a sample size but randomly sampled a large population with the same age and gender profile as the general population. 

The questionnaire was split into two parts and is presented in full in Appendix A. The first 18 questions collected data on basic demographics (e.g., age, sex, area of residence, and access to healthcare), the most recent virtual appointment (e.g., who it was for, how long ago, and what the consultation was for), how the appointment was provided (e.g., Call 973, Sehhaty app, Seha virtual hospital, other government hospital, or private hospital), and how the consultation was performed. 

The second part of the questionnaire consisted of 37 questions about opinions on telehealth service delivery and technology with respect to the respondent’s most recent virtual appointment. The questionnaire was based on the Telehealth Usability Questionnaire (TUQ) [30], which was designed to capture information about technology implementation and services in six domains: usefulness and convenience (ten questions), ease of use (seven questions), effectiveness (seven questions), reliability (three questions), quality of the interaction (five questions), and satisfaction (five questions). Answers to all questions were in the form of a 7-point Likert scale where 1 = strongly disagree, 2 = disagree, 3 = somewhat disagree, 4 = neither agree nor disagree, 5 = somewhat agree, 6 = agree, and 7 = strongly agree.

Statistical analyses were performed in SPSS v29 (IBM Statistics, Armonk, NY, USA). Most data were categorical variables and are presented as counts and percentages. Age is presented as mean (SD). Responses to questions about opinions on the respondent’s most recent virtual appointment were dichotomized into “agree” (somewhat agree, agree, and strongly agree) or “neutral or disagree” (strongly disagree, disagree, somewhat disagree, and neither agree nor disagree). To examine potential associations between negative opinion scores and demographic characteristics, we calculated a total opinion score by summing all Likert responses and considered those with scores in the bottom quartile as “less happy” and those in the other three quartiles as “happier” with their virtual consultation. Associations between demographic variables and these categorizations were assessed with the chi-squared test (Student’s *t*-test for age). A *p*-value of <0.05 was considered significant.

## 3. Results

The participant demographics are shown in Table 1. Indeed, 492/916 (53.7%) participants were female, with a mean age of 47.2 (14.1) years. Nearly three-quarters were married, and the largest proportion lived in the central part of the KSA (38.9%), followed by the western areas (21.0%). The majority of the respondents lived in rural areas (69.0%), with a similar proportion living >100 km from the nearest hospital.

When asked to recall their most recent virtual appointment, 83.8% considered appointments within the last six months (Table 2). A similar proportion had attended for themselves rather than for a child or family member. Nearly half (45.0%) attended primary care appointments, with the remainder attending a range of hospital specialties, the most common being diabetes and endocrinology (12.7%); nearly all saw physicians (95.6%). A third attended cardiovascular appointments or diabetes checks (304/916, 33.2%), with the next most common reason for attendance general checkups or smoking cessation activities (260/916, 28.4%) followed by respiratory problems (8.7%), allergies and asthma (6.1%), and sleeping problems (6.1%). About 40% each were attending as new patients and routine followup, respectively, with smaller numbers seeking medication refills (13.1%) or test results (6.1%).

There was an approximately equal provision of appointments from government and private hospitals (about a third each), with 12.7% accessing appointments via the Sehhaty National Population Health Platform app (Table 2). Over 90% preferred having a virtual appointment over an in-person visit, and a similar proportion had actively chosen a virtual appointment. About half had telephone consultations, while about a third had video calls through hospital-provided platforms, and about half of the respondents were using a remote consultation system for the first time.

For nearly all the opinion questions (Table 3), >90% found their virtual appointments to be useful and convenient, easy to use, effective, reliable, and to produce a favorable clinical interaction. The respondents were highly satisfied with their virtual consultations. While one in four respondents experienced technical difficulties during their appointment, nearly all (97.4%) were nevertheless satisfied with their remote consultation experience, and 96.5% would recommend the service to friends and family. 

Finally, and although relatively few individuals expressed negative opinions about their virtual consultations, we examined any potential associations between more negative opinion scores (those with the total opinion scores in the bottom quartile) and demographic characteristics (Table 4). Overall, the individuals who were less happy with their virtual consultation were significantly younger, lived in urban areas close to hospitals, were attending specialty clinics (especially psychiatry), used the Sehhaty app, were seen by a psychologist, already had a preference to be seen in person, and had consultations by telephone.

## 4. Discussion

This is one of the largest population-wide cross-sectional studies conducted to date on opinions about telehealth across the KSA and elsewhere. Our analysis of over 900 individuals, primarily living rurally and attending virtual appointments by telephone and video call for a range of health conditions in primary and secondary care, revealed that over nine in ten participants found their virtual appointments to be useful and convenient, easy to use, effective, reliable, and produce a favorable clinical interaction. The respondents were highly satisfied with their virtual consultations despite a relatively high occurrence of technical difficulties during the interaction, occurring in a fifth of the cases. Although a few participants expressed negative opinions about virtual health appointments, this population tended to be younger, lived in urban settings, attended specialty clinics (especially psychiatry), used the Sehhaty app, already preferred in-person consultations, and had consultations by telephone. Over an eighteen-month period in 2021 and 2022, over a million virtual consultations were delivered to the population of the KSA [9]; assuming that the virtual consultations remained constant after this time (i.e., ~55,000 virtual consultations a month), our sample represents about 0.5% of the population attending virtual consultations. Given this sample size and that the age (relatively young) and sex profile (female predominance) reflected that of the individuals seeking virtual consultations from population-level administrative data [9], our results are likely to be representative of the overall population attending virtual consultations.

Although until now no study has comprehensively evaluated all the domains of usefulness, convenience, effectiveness, reliability, and satisfaction of virtual consultations, the favorable results are consistent with several recent studies and meta-analyses on patient satisfaction with telehealth [18,19,20,29]. A very recent meta-analysis of over a hundred cross-sectional studies from around the world on patient satisfaction with telemedicine similarly reported very high patient satisfaction, with less than 3% reporting satisfaction levels below 75% [18]. The recent COVID-19 pandemic prompted several studies on patient satisfaction with telemedicine in Saudi Arabia. For instance, Abdel Nasser at al. [19] surveyed 425 patients treated through telemedicine programs in the KSA in 2020 and found that ~80% of the participants were either very satisfied or satisfied with various domains analogous to those examined in our study: registration/scheduling (ease of use), the quality of the visual image and audio (effectiveness), the ability to understand the recommendations or diagnosis made (interaction), and the overall quality of the care and telemedicine experience (satisfaction). In another questionnaire-based survey of 641 patients who attended primary medicine telemedicine clinics in Jeddah [20], 82.7% of the patients were satisfied with the telemedicine services. Other studies from the KSA have similarly reported high satisfaction with telemedicine in both general [23,24,25] and specialist [26,27,28] practice.

Understanding the factors influencing opinions about telemedicine can help healthcare providers to improve telemedicine services, patient–provider relationships, and healthcare delivery. Few participants expressed negative opinions about virtual health appointments in the current study, and those that did tended to be younger, lived in urban settings close to hospitals, attended specialty clinics (especially psychiatry), used the Sehhaty app (3.10.0), already had a preference for in-person consultations, and had consultations by telephone. This profile reflects the complex relationship between patients’ acceptance of telehealth services and attitudes towards healthcare services, personal circumstances, and the nature of the clinical interactions for different specialties. For instance, mirroring the positive opinions about telehealth consultations found here in individuals preferring this type of consultation, Abdel Nasser et al. [19] reported a significant positive correlation between satisfaction and attitudes towards telehealth, and Alshahrani et al. [32] found that in-person consultation preference was the largest barrier preventing Saudis from using telemedicine services. Similarly, a meta-analysis [18] also revealed that the time interval between the consultation and satisfaction assessment influenced the satisfaction levels, as in this study. Telemedicine is likely to facilitate access to healthcare provision in individuals in rural settings living distant from secondary and tertiary services, and the inconvenience and cost of traveling to healthcare appointments is a known barrier to healthcare provision [25], thereby enhancing the satisfaction with telehealth when it is available. Interestingly, Abdulwahab et al. [24] found that the satisfaction with telemedicine services was significantly higher in specific specialties (cardiology and orthopedics) and that the telemedicine services were unpopular in psychiatry clinics, possibly reflecting the greater emphasis placed on patient–therapist relationships in mental health provision and their positive influence on the outcomes [33]. Although most of the other studies reported older age as a negative predictor of satisfaction with telehealth [20,25,34], our results only reflected a small age difference of approximately two years (i.e., the gap did not reflect a major generational difference), which is of uncertain significance. Overall, therefore, our data highlight the importance of telehealth provision in rural areas and the need to enhance positive attitudes towards telehealth, such as through encouraging performance and effort expectancy (i.e., the degree to which an individual believes that using telemedicine could be helpful and the ease with which it is accessed), social influence (ensuring that the individual believes that others think that they should use telemedicine), and by reducing the barriers to its use (through technology and infrastructure). With respect to the latter, the use of the Sehhaty app was associated with negative opinions about telehealth, and the reasons for this require further exploration.

This study has limitations. As noted above, convenience sampling is inherently biased, and the results relate directly to the study population; nevertheless, this was a relatively large sample with an age and sex profile (female predominance) reflecting that of those seeking virtual consultations from the population-level administrative data [9], thus potentiating that the results are more likely to be generalizable to the wider population. Three-quarters of the population of the KSA are aged between 15 and 74 years [1,2]; that is, the population is relatively young, so, given that older people might face increased barriers to technology use [35], the results may not be generalizable to countries with older populations. Although questionnaire studies are subject to recall bias, the appointments were within the last six months in 80% of the cases. Some unmeasured parameters, such as the ease of appointment scheduling, mobile signal type and strength, health insurance coverage, and educational status, may have also impacted the results; future studies should include these factors. Finally, although we based the questionnaire on the TUQ [30], which was designed to capture information about perspectives on technology implementation and services across several important telehealth use domains, we added additional questions, meaning that the final version was not fully validated. As noted in a recent meta-analysis [18], there is a need for standardized measurement instruments for telemedicine satisfaction and use assessment to ensure reliability, generalizability, and comparability.

## 5. Conclusions

Nevertheless, this is one of the largest studies concerning opinions about telehealth conducted to date, and the data provide momentum and reassurance to continue with and expand telehealth services, supported by initiatives to further provide education regarding its advantages. Our results show that the users were highly satisfied with their virtual consultations despite a relatively high occurrence of technical difficulties during the interaction, occurring in a fifth of the cases. Although a few participants expressed negative opinions about virtual health appointments, this population tended to be younger, lived in urban settings, attended specialty clinics (especially psychiatry), used the Sehhaty app, already preferred in-person consultations, and had consultations by telephone. The future developments in telehealth in the KSA need to overcome individual, cultural, and technological barriers. For instance, the future initiatives should ensure that the convenience and flexibility of the digital health platforms resonate with the lifestyles and expectations of specific demographic groups, especially the urban young. Any cultural resistance to telehealth might be addressed through public health training and education strategies, such as the distribution of brochures or manuals on telehealth and its advantages. Our data might suggest that specific apps, such as Sehhaty, require a redesign to ensure that they are user-friendly and effective. Finally, given that only a third of the participants used video calling but in general this consultation modality was preferred, telehealth provision must prioritise this mode of communication. These changes and developments could be supported by action research projects aimed at improving telehealth services based on patient feedback and stakeholder engagement in Saudi Arabia.

## Figures and Tables

**Table 1:** Participant demographics.

Characteristic		Number	%
Sex	Male	424	46.3
	Female	492	53.7
Age (mean, SD)		47.2, 14.1	
Relationship status	Married	667	72.8
	Not married	249	27.3
Area of residence	Central	356	38.9
	Eastern	148	16.2
	Northern	116	12.7
	Southern	104	11.4
	Western	192	21.0
Urban or rural	Urban	284	31.0
	Rural	632	69.0
Distance from nearest hospital	<50 km	272	29.7
	50–100 km	108	11.8
	100–300 km	268	29.3
	>300 km	248	27.1
	Unsure	20	2.2

**Table 2:** General appointment characteristics.

Characteristic		Number	%
Time since most recent appointment	<3 months	436	47.6
	3–6 months	332	36.2
	7–9 months	76	8.3
	10–12 months	48	5.2
	>12 months	24	2.6
Appointment patient	Participant	804	87.8
	Someone else (e.g., child, family member)	112	12.2
Department	Allergy and immunology	0	0.0
	Cardiology	44	4.8
	Dermatology	8	0.9
	Diabetes and endocrinology	116	12.7
	Emergency	0	0.0
	ENT	40	4.4
	Gastroenterology	20	2.2
	General surgery	0	0
	Hematology	0	0.0
	Infectious diseases	0	0.0
	Nephrology	24	2.6
	Neurology	60	6.6
	Obstetrics and gynecology	12	1.3
	Oncology	0	0.0
	Ophthalmology	4	0.4
	Pediatrics	36	3.9
	Primary care	412	45.0
	Psychiatry	68	7.4
	Respiratory medicine	40	4.4
	Rheumatology	8	0.9
	Sleep medicine	8	0.8
	Smoking cessation	12	1.3
	Urology	4	0.4
Reason for attendance	Allergy (including asthma)	56	6.1
	Arthritis, joint and back pain	16	1.7
	Neurology, including headaches	28	3.1
	Respiratory problems (excluding asthma)	80	8.7
	Psychological or psychiatric conditions	52	5.7
	Cardiovascular disease, including diabetes	304	33.2
	Dermatological conditions	16	1.7
	Pediatrics	8	0.9
	Gastrointestinal conditions	16	1.7
	Sleep problems, including OSA	56	6.1
	Obesity	8	0.9
	Other, including general health check-up or smoking cessation	260	28.4
	Peri- or postnatal care	8	0.9
	Renal	8	0.9
Who provided the appointment?	Call 973	28	3.1
	Other government hospital	344	37.6
	Other private healthcare app	72	7.9
	Private hospital	324	35.4
	Seha virtual hospital	32	3.5
	Sehhaty app	116	12.7
Healthcare professional seen	Doctor	876	95.6
	Nurse	8	0.9
	Psychologist	24	2.6
	Don’t know	8	0.9
New appointment or for pre-existing condition	New consultation	376	41.0
	Routine follow-up	360	39.3
	Followup for results	56	6.1
	Follow-up for medication refill	120	13.1
	Missing	4	0.4
Preferred type of appointment	In-person	40	4.4
	Virtual	848	92.8
	Missing	28	3.1
How was the virtual consultation performed?	By audio call through a hospital-provided platform	76	8.3
	By telephone	476	52.0
	By text, email, or messaging platform	28	3.1
	By video call through a hospital-provided platform	280	30.6
	By video call through a commercial platform (e.g., Teams, Zoom, etc.)	24	2.6
	Don’t know/unsure	32	3.5
Was this the first time using this remote consultation system?	No	444	48.5
	Yes	472	51.5
My remote consultation visit was my choice	No	128	14.0
	Yes	788	86.0

**Table 3:** Usefulness and convenience, usability, effectiveness, reliability, and satisfaction of virtual consultations.

Question	Response	Number	%
**USEFULNESS AND CONVENIENCE**			
Compared with an in-person visit, the remote consultation improved my access to healthcare services	Neutral or disagree	24	2.6
	Agree	892	97.4
The remote consultation was more convenient for me than an in-person visit due to my health status (e.g., difficulty in moving, age, disabilities, etc.)	Neutral or disagree	76	8.3
	Agree	840	91.7
The remote consultation was more convenient for me than an in-person visit due to health safety reasons (e.g., avoiding hospital infections)	Neutral or disagree	88	9.6
	Agree	828	90.4
The remote consultation was convenient for me because of the distance I need to travel to visit the healthcare professional	Neutral or disagree	56	6.1
	Agree	860	93.9
The remote consultation (compared with an in-person visit) saved me time traveling to a hospital or specialist clinic	Neutral or disagree	84	9.2
	Agree	832	90.8
The remote consultation was convenient for me because it provided timely access to a healthcare professional	Neutral or disagree	24	2.6
	Agree	892	97.4
The remote consultation provided me with access to a medical subspeciality not available locally	Neutral or disagree	104	11.4
	Agree	812	88.6
The remote consultation was convenient for me because I need frequent (e.g., monthly) visits.	Neutral or disagree	92	10.0
	Agree	824	90.0
The remote consultation provided for my healthcare need	Neutral or disagree	40	4.4
	Agree	876	95.6
Overall, I found the remote consultation very convenient	Neutral or disagree	28	3.1
	Agree	888	96.9
**EASE OF USE**			
It was simple to schedule an appointment with the remote consultation system	Neutral or disagree	24	2.6
	Agree	892	97.4
It was simple to use the remote consultation system	Neutral or disagree	16	1.7
	Agree	900	98.3
It was simple to learn to use the remote consultation system	Neutral or disagree	20	2.2
	Agree	896	97.8
I believe I could become productive quickly using the remote consultation system	Neutral or disagree	28	3.1
	Agree	888	96.9
The way I interacted with the remote consultation system was pleasant	Neutral or disagree	32	3.5
	Agree	884	96.5
I liked using the remote consultation system	Neutral or disagree	28	3.1
	Agree	888	96.9
The remote consultation system was simple and easy to understand	Neutral or disagree	16	1.7
	Agree	900	98.3
**EFFECTIVENESS**			
The remote consultation system was able to do everything I would want it to be able to do	Neutral or disagree	36	3.9
	Agree	880	96.1
I could easily communicate with the healthcare professional using the remote consultation system	Neutral or disagree	28	3.1
	Agree	888	96.9
I experienced technical difficulties during the remote consultation	Neutral or disagree	728	79.5
	Agree	188	20.5
I could recover the consultation easily and quickly when the technology failed	Neutral or disagree	76	8.3
	Agree	840	91.7
I could hear the clinician clearly using the remote consultation system	Neutral or disagree	24	2.6
	Agree	892	97.4
I felt I was able to express myself effectively	Neutral or disagree	32	3.5
	Agree	884	96.5
Using the remote consultation system, I could see the clinician as well as if we met in person	Neutral or disagree	308	33.6
	Agree	608	66.4
**RELIABILITY**			
I think the visit provided over the remote consultation system was the same as in-person visits	Neutral or disagree	52	5.7
	Agree	864	94.3
Whenever I made a mistake using the system, I could recover easily and quickly	Neutral or disagree	80	8.7
	Agree	836	91.3
The system gave error messages that clearly told me how to fix problems	Neutral or disagree	108	11.8
	Agree	808	88.2
**INTERACTION**			
My virtual consultation visit started on time	Neutral or disagree	44	4.8
	Agree	872	95.2
My provider explained how my confidentiality is protected	Neutral or disagree	76	8.3
	Agree	840	91.7
My privacy was respected	Neutral or disagree	32	3.5
	Agree	884	96.5
My questions about the technology used during my virtual consultation were answered	Neutral or disagree	84	9.2
	Agree	832	90.8
My healthcare provider explained things in a way that was easy to understand	Neutral or disagree	32	3.5
	Agree	884	96.5
**SATISFACTION**			
I felt comfortable communicating with the healthcare professional using the remote consultation system	Neutral or disagree	32	3.5
	Agree	884	96.5
Remote consultation is an acceptable way to receive healthcare services	Neutral or disagree	32	3.5
	Agree	884	96.5
I would use remote consultation services again	Neutral or disagree	28	3.1
	Agree	888	96.9
I would recommend remote consultations to family and friends	Neutral or disagree	32	3.5
	Agree	884	96.5
Overall, I was satisfied with this remote consultation system	Neutral or disagree	24	2.6
	Agree	892	97.4

**Table 4:** Associations between low total virtual consultation opinion scores (cutoff 25th percentile) and baseline parameters.

Characteristic		Less Happy with Virtual Consultation	Happier with Virtual Consultation	*p*-Value
Sex	Male	121 (51.3)	371 (54.6)	0.405
	Female	115 (48.7)	309 (45.4)	
Age (mean, SD)		45.3 (11.8)	47.8 (14.7)	<0.001
Marriage status	Married	176 (74.6)	491 (72.2)	0.498
	Not married	60 (25.4)	189 (27.8)	
Rural/urban	Urban	100 (42.4)	184 (27.1)	<0.001
	Rural	136 (57.6)	496 (72.9)	
Distance from nearest hospital	<50 km	96 (40.7)	176 (25.9)	<0.001
	50–100 km	16 (6.8)	92 (13.5)	
	100–300 km	68 (28.8)	200 (29.4)	
	>300 km	48 (20.3)	200 (29.4)	
	Unsure	8 (3.4)	12 (1.8)	
Time since last appointment	<3 months	100 (42.4)	336 (49.4)	0.006
	3–6 months	96 (40.7)	236 (34.7)	
	7–9 months	12 (5.1)	64 (9.4)	
	10–12 months	20 (8.5)	28 (4.1)	
	>12 months	8 (3.4)	16 (2.4)	
Appointment patient	Participant	200 (84.7)	604 (88.8)	0.107
	Someone else (e.g., child, family member)	36 (15.3)	76 (11.2)	
Department	Cardiology	4 (1.7)	40 (5.9)	<0.001
	Dermatology	0 (0.0)	8 (1.2)	
	Diabetes and endocrinology	16 (6.8)	100 (14.7)	
	ENT	28 (11.9)	12 (1.8)	
	Gastroenterology	12 (5.1)	8 (1.2)	
	Nephrology	8 (3.4)	16 (2.4)	
	Neurology	12 (5.1)	48 (7.1)	
	Obstetrics and gynecology	12 (5.1)	0 (0.0)	
	Ophthalmology	0 (0.0)	4 (0.6)	
	Pediatrics	12 (5.1)	24 (3.5)	
	Primary care	76 (32.2)	336 (49.4)	
	Psychiatry	28 (11.9)	40 (5.9)	
	Respiratory medicine	20 (8.5)	20 (2.9)	
	Rheumatology	4 (1.7)	4 (0.6)	
	Sleep medicine	0 (0.0)	8 (1.2)	
	Smoking cessation	4 (1.7)	8 (1.2)	
	Urology	0 (0.0)	4 (0.6)	
Who provided the appointment?	Call 973	8 (3.4)	20 (2.9)	<0.001
	Other government hospital	92 (39.0)	252 (37.1)	
	Other private healthcare app	16 (6.8)	56 (8.2)	
	Private hospital	72 (30.5)	252 (37.1)	
	Seha virtual hospital	0 (0.0)	32 (4.7)	
	Sehhaty app	48 (20.3)	68 (10.0)	
Healthcare professional seen	Doctor	224 (94.9)	652 (95.9)	0.005
	Nurse	0 (0.0)	8 (1.2)	
	Psychologist	12 (5.1)	12 (1.8)	
	Don’t know	0 (0.0)	8 (1.2)	
New appointment or for pre-existing condition	New consultation	92 (39.0)	284 (41.8)	0.113
	Routine follow-up	100 (42.4)	260 (38.2)	
	Follow-up for results	20 (8.5)	36 (5.3)	
	Follow-up for medication refill	24 (10.2)	96 (14.1)	
	Missing	0 (0.0)	4 (0.6)	
Preferred type of appointment	In-person	24 (10.2)	16 (2.4)	<0.001
	Virtual	200 (84.7)	648 (95.3)	
	Missing	12 (5.1)	16 (2.4)	
How was the virtual consultation performed?	By audio call through a hospital-provided platform	28 (11.9)	48 (7.1)	<0.001
	By telephone	136 (57.6)	340 (50.0)	
	By text, email, or messaging platform	12 (5.1)	16 (2.4)	
	By video call through a hospital-provided platform	0 (0.0)	24 (3.5)	
	By video call through a commercial platform (e.g., Teams, Zoom, etc.)	48 (20.3)	232 (34.1)	
	Don’t know/unsure	12 (5.1)	20 (2.9)	

## Data Availability

The data supporting the study findings can be obtained from the corresponding author on reasonable request.

## Appendix A. Questionnaire on Telehealth Use, Convenience, Effectiveness, Reliability, and Satisfaction in Saudi Arabia

New technologies are changing the way in which we interact with our healthcare providers, and telephone or video consultations are becoming more common. We want to know how patients feel about these changes to the way in which they see healthcare professionals.

1. Are you male or female?

Options: male, female

2. How old are you?

Option: free text, number

3. Are you married/widowed, separated/divorced, never married?

Options: married/widowed, separated/divorced, never married

4. Which part of the country do you live in?

Options: central/eastern/western/northern/southern

5. Do you live in a city or in the countryside?

Options: city, countryside

6. How far are you from your nearest hospital?

(<50 km, 50–100 km, 100–300 km, >300 km, I don’t know)

7. Approximately how long ago was the appointment (in months)?

Option: less than three months, from 3–6 months, from 7–9 months, from 10–12 months, more than a year and after a period of COVID restrictions, during a period of COVID restrictions.

8. Was the appointment for you or with someone else (i.e., a child, family member)

Options: for myself, for someone else

9. Which department was your appointment with?

Options: primary care (family/general medicine), medicine (cardiology, respiratory medicine, nephrology, diabetes and endocrinology, allergy and immunology, neurology, rheumatology, infectious disease, hematology, oncology, gastroenterology, psychiatry, psychology and psychotherapy, smoking cession, dermatology, sleep medicine), surgery (general surgery, ENT, ophthalmology, urology, orthopedics), pediatrics, obstetrics and gynecology, emergency

10. Why did you see the healthcare professional?

Options: hypertension, hyperlipidemia, arthritis & joint disorders, diabetes, depression or anxiety, obesity, asthma, allergic rhinitis and or allergic sinusitis, cancer, COPD, osteoporosis, skin disorders, back problems, upper respiratory infections, prenatal or post-natal care, chronic neurologic disorders, headaches and migraines, GERD, irritable bowel syndrome, obstructive sleep apnea, insomnia, other sleep disorder, psychotherapy, smoking cessation, periodic health examination, other (free text)

11. Was this your first consultation for this complaint? Options: yes/no

12. Who provided the appointment?

Options: Call 973, Sehhaty app, Seha virtual hospital, other government hospital, private hospital, Other private healthcare apps like Cura, Vezeeta, labayh. etc., I can’t remember.

13. What type of healthcare professional did you see?

Options: doctor, nurse, pharmacist, physiotherapist, psychologist, occupational therapist, can’t remember/don’t know

14. Was your appointment a new consultation or for a pre-existing health problem?

Options: new consultation, routine follow-up for ongoing health problem, follow-up for results, follow-up for medication re-fill

15. For healthcare consultations, which type you prefer?

Virtual consultation, onsite consultation

16. How was the virtual consultation performed?

Options: by telephone, by audio call through a hospital-provided platform, by video call through a hospital-provided platform, by video call through a commercial platform (e.g., Teams, Zoom, etc.), by text/email/messaging platform, don’t know/unsure.

17. Was this the first time using this remote consultation system?

Options: yes/no

18. My remote consultation visit was my choice

Options: yes/no

all the following are on a 7-point likert scale where 1—strongly disagree, 2—disagree, 3—somewhat disagree, 4—neither agree nor disagree, 5—somewhat agree, 6—agree, 7—strongly agree, n/a

19. The remote consultation (compared with an in-person visit) improved my access to healthcare services (usefulness/convenience)

20. The remote consultation was more convenient for me than an in-person visit due to my health status (e.g., difficulty in moving, age, disabilities, etc.) (usefulness/convenience)

21. The remote consultation was more convenient for me than an in-person visit due to health safety reasons (e.g., avoiding hospital infections) (usefulness/convenience)

22. The remote consultation was convenient for me because of the distance I need to travel to visit the healthcare professional (usefulness/convenience)

23. The remote consultation (compared with an in-person visit) saved me time traveling to a hospital or specialist clinic (usefulness/convenience)

24. The remote consultation was convenient for me because it provided timely access to a healthcare professional (usefulness/convenience)

25. The remote consultation provided me with access to a medical subspeciality not available locally (usefulness/convenience)

26. The remote consultation was convenient for me because I need frequent (e.g., monthly) visits (usefulness/convenience)

27. The remote consultation provided for my healthcare need (usefulness/convenience)

28. Overall, I found the remote consultation very convenient (usefulness/convenience)

29. It was simple to schedule an appointment with the remote consultation system (ease of use)

30. It was simple to use the remote consultation system (ease of use)

31. It was simple to learn to use the remote consultation system (ease of use)

32. I believe I could become productive quickly using the remote consultation system (ease of use)

33. The way I interacted with the remote consultation system was pleasant (ease of use)

34. I liked using the remote consultation system (ease of use)

35. The remote consultation system was simple and easy to understand (ease of use)

36. The remote consultation system was able to do everything I would want it to be able to do (effectiveness)

37. I could easily communicate with the healthcare professional using the remote consultation system (effectiveness)

38. I experienced technical difficulties during the remote consultation (effectiveness)

39. I could recover the consultation easily and quickly when the technology failed (effectiveness)

40. I could hear the clinician clearly using the remote consultation system (effectiveness)

41. I felt I was able to express myself effectively (effectiveness)

42. Using the remote consultation system, I could see the clinician as well as if we met in person (effectiveness)

43. I think the visit provided over the remote consultation system was the same as in-person visits (reliability)

44. Whenever I made a mistake using the system, I could recover easily and quickly (reliability)

45. The system gave error messages that clearly told me how to fix problems (reliability)

46. My virtual consultation visit started on time (interaction)

47. My provider explained how my confidentiality is protected (interaction)

48. My privacy was respected (interaction)

49. My questions about the technology used during my virtual consultation were answered (interaction)

50. My healthcare provider explained things in a way that was easy to understand (interaction)

51. I felt comfortable communicating with the healthcare professional using the remote consultation system (satisfaction)

52. Remote consultation is an acceptable way to receive healthcare services (satisfaction)

53. I would use remote consultation services again (satisfaction)

54. I would recommend remote consultations to family and friends (satisfaction)

55. Overall, I was satisfied with this remote consultation system (satisfaction).
